# Finding Important Terms for Patients in Their Electronic Health Records: A Learning-to-Rank Approach Using Expert Annotations

**DOI:** 10.2196/medinform.6373

**Published:** 2016-11-30

**Authors:** Jinying Chen, Jiaping Zheng, Hong Yu

**Affiliations:** ^1^ Department of Quantitative Health Sciences University of Massachusetts Medical School Worcester, MA United States; ^2^ School of Computer Science University of Massachusetts Amherst, MA United States; ^3^ Bedford Veterans Affairs Medical Center Center for Healthcare Organization and Implementation Research Bedford, MA United States

**Keywords:** electronic health records, natural language processing, information extraction, supervised learning, learning to rank

## Abstract

**Background:**

Many health organizations allow patients to access their own electronic health record (EHR) notes through online patient portals as a way to enhance patient-centered care. However, EHR notes are typically long and contain abundant medical jargon that can be difficult for patients to understand. In addition, many medical terms in patients’ notes are not directly related to their health care needs. One way to help patients better comprehend their own notes is to reduce information overload and help them focus on medical terms that matter most to them. Interventions can then be developed by giving them targeted education to improve their EHR comprehension and the quality of care.

**Objective:**

We aimed to develop a supervised natural language processing (NLP) system called Finding impOrtant medical Concepts most Useful to patientS (FOCUS) that automatically identifies and ranks medical terms in EHR notes based on their importance to the patients.

**Methods:**

First, we built an expert-annotated corpus. For each EHR note, 2 physicians independently identified medical terms important to the patient. Using the physicians’ agreement as the gold standard, we developed and evaluated FOCUS. FOCUS first identifies candidate terms from each EHR note using MetaMap and then ranks the terms using a support vector machine-based learn-to-rank algorithm. We explored rich learning features, including distributed word representation, Unified Medical Language System semantic type, topic features, and features derived from consumer health vocabulary. We compared FOCUS with 2 strong baseline NLP systems.

**Results:**

Physicians annotated 90 EHR notes and identified a mean of 9 (SD 5) important terms per note. The Cohen’s kappa annotation agreement was .51. The 10-fold cross-validation results show that FOCUS achieved an area under the receiver operating characteristic curve (AUC-ROC) of 0.940 for ranking candidate terms from EHR notes to identify important terms. When including term identification, the performance of FOCUS for identifying important terms from EHR notes was 0.866 AUC-ROC. Both performance scores significantly exceeded the corresponding baseline system scores (*P*<.001). Rich learning features contributed to FOCUS’s performance substantially.

**Conclusions:**

FOCUS can automatically rank terms from EHR notes based on their importance to patients. It may help develop future interventions that improve quality of care.

## Introduction

### Background and Significance

Greater patient involvement is indispensable in delivering high-quality patient-centered care. In one effort to achieve this goal, spurred by the Health Information Technology for Economic and Clinical Health Act [1,2] and the Centers for Medicare and Medicaid Services Medicare Electronic Health Record (EHR) incentive program [3], online patient portals have been widely adopted by health systems in the United States [3,4]. In addition to giving patients structured information from EHRs (eg, laboratory test results and medication lists), the OpenNotes initiative [5] and the Blue Button movement [6] allow patients to access their full EHR notes through patient portals. Early evidence shows improved medical comprehension, health care management, and outcomes from the OpenNotes initiative [7-9].

However, the benefits from accessing their full EHR notes would be compromised if patients cannot comprehend their notes. EHRs were created for physician-physician communication, and thus are frequently long and contain abundant medical jargon. Patients who usually do not have the same medical training as physicians are likely overwhelmed by the medical jargon, and therefore face an enormous challenge in comprehending their notes. For example, EHRs were written at an 8^th^-12^th^-grade reading level [10-13], which is above the average adult patient’s reading level of 7^th^-8^th^grade in the United States [14-19]. In addition, 36% of adult Americans have limited health literacy [19] and have shown difficulty in comprehending medical jargon [20-25]. In fact, limited health literacy has been identified as one of the major barriers to patient online portal use, which includes the interpretation of information from EHRs [26-28]. Therefore, information technologies that support EHR comprehension are much needed to supplement the widespread use of patient portals and EHRs among patients.

To support patient EHR comprehension, this work focuses on identifying medical terms that matter most to individual patients in their EHR notes—we used the 2 phrases “medical terms” and “medical jargon” interchangeably in this paper. Our work was motivated by 2 reasons. First, medical terms, which are fundamental to discourse-level EHR comprehension, have been shown to be obstacles for patients [20-25]. Second, EHR notes incorporate a comprehensive description of patients’ medical courses yet patients may care about their immediate concerns. For example, a radiology report may describe technical details of tumor images; however, the patient may want to know only the tumor size, the diagnosis, and the prognosis. When helping patients comprehend their own EHR notes, the approach of explaining all the jargon in their notes may likely overwhelm them and may be unnecessary in the first place.

Therefore, in this study we identify medical jargon most important to individual patients. Personalized interventions can then be developed by giving targeted educational materials to each individual patient.

In order to find out whether medical terms can be prioritized, we asked physicians to identify terms important to patients in EHRs. Textbox 1 shows an excerpt from a typical EHR note from our corpus. Although there are many medical terms in this piece of text—here we only highlighted a subset of terms identified by MetaMap [29] for illustration purposes—physicians identified only 5 terms most important for patients to know: *thrombocytosis*, *Crohn disease*, *budesonide*, *diabetes mellitus*, and *metformin*. Note that physicians do not mark many unfamiliar medical terms (eg, *complete blood count* [*CBC*], *hematemesis*, and *epistaxis*), suggesting that they do not rank terms based on their difficulty levels.

**A sample electronic health record text where physicians identified important medical terms (bracketed with angle brackets). Other medical terms are italicized.**

xxx is a xx-year-old man referred for evaluation of <thrombocytosis>. Prior *CBCs* from xxx through xxx revealed *platelet counts* ranging from 400,000 to 500,000, but no more recent studies are available. He has long-standing <Crohn disease> and although he says he has not had *gastrointestinal bleeding* in the past, he has been given iron, which he is taking twice daily. He has black stool, but notes no blood and he has not had *hematemesis*. He notes no blood in his urine or sputum and he has no *epistaxis*. He discontinued the use of iron yesterday because he thought that might alleviate his gastrointestinal complaints, but he does not feel different today. He is cared for by Dr. xxx at xxx Hospital Medical Center in xxx. He has no history of prior cancers, *tuberculosis* or other infectious diseases. He has been taking <budesonide> for his <Crohn disease>. He has no unexplained fevers, although he states he often feels hot. He has no soaking sweats and has not had unexplained weight loss. He believes he was referred to an *oncologist* many years ago at xxx, but he cannot recall the reason for that referral, who the doctor was, or what the findings were. He often feels queasy and nauseated, but has no vomiting. He has loose stools up to 4 days per week, but has had a stable pattern of <Crohn disease>. Also notable for <diabetes mellitus> for which he takes <metformin> and has required no *insulin* and has had no complications of *retinopathy* or *renal dysfunction*. <Crohn disease> as described above and an enlarged prostate.

Our aim was to develop a supervised natural language processing (NLP) system called Finding impOrtant medical Concepts most Useful to patientS (FOCUS) to automatically rank those EHR (patient)-specific important terms as high. This task was challenging, as the problem could not be solved by using only simple strategies such as term unfamiliarity, term frequency, and handcrafted rules (details in the Discussion section). We therefore built FOCUS with supervised learning and rich features.

To the best of our knowledge, our work is the first to successfully rank medical terms in EHR notes by focusing on patients’ needs. This is an important step toward information reduction and personalized interventions to improve patient EHR comprehension. Our contributions are multifold. First, we defined a new NLP task of prioritizing or ranking medical terms that are important for patients. Second, we developed a state-of-the-art learning-based NLP system to automate the task. Third, we explored novel semantically motivated learning features.

By using a robust learning framework, FOCUS can be readily adapted to other NLP tasks including summarization and question answering.

### Related Works

#### Natural Language Processing Systems Facilitating Concept-Level Electronic Health Record Comprehension

There has been active research on linking medical terms to lay terms [11,30,31], consumer-oriented definitions [12] and educational materials [32], and showing improved comprehension with such interventions [11,12].

On the issue of determining which medical terms to simplify, there is previous work that used frequency-based and/or context-based approaches to check if a term is unfamiliar to the average patient or if it has simpler synonyms [11,30,31]. Such work focuses on identifying difficult medical terms and treats these terms as equally important.

Our approach is different in 2 aspects: (1) we focus on finding important medical terms, which are not equivalent to difficult medical terms, as discussed in the Background and Significance subsection; and (2) our approach is patient centered and prioritizes important terms for each EHR note of individual patients. We developed several learning features, including term frequency, term position, term frequency-inverse document frequency (TF-IDF), and topic feature, to serve this purpose.

It is worth noting that our approach is complementary to previous work. For example, in a real-world application, we can display the lay definitions for all the difficult medical terms in a patient’s EHR note, and then highlight those terms that FOCUS predicts to be most important to this patient.

#### Single-Document Keyphrase Extraction

Our work is inspired by, but different from, single-document keyphrase extraction (KE), which identifies terms or phrases representing important concepts and topics in a document. KE targets topics that the writers wanted to convey when writing the documents. Unlike KE, our work does not focus on topics important to physicians (ie, the writers and the target readers when writing the EHR notes), but rather focuses on patients, the new readers of the notes.

Both supervised and unsupervised methods have been developed for KE [33]. We use supervised methods, which in general perform better than unsupervised ones when training data is available.

Most supervised methods formulate KE as a binary classification problem. The confidence scores output by the classification algorithms are used to rank candidate phrases. Various algorithms have been explored, such as naïve Bayes, decision tree, bagging, support vector machine (SVM), multilayer perceptron, and random forest (RF) [34-43]. In our study, we implemented RF [43] as a strong baseline system.

KE in the biomedical domain mainly focused on literature articles and domain-specific methods and features [44-47]. For example, Li et al [44] developed a software tool called keyphrase identification program (KIP) to extract keyphrases from medical articles. KIP used Medical Subject Headings (MeSH) as the knowledge base to compute a score to reflect a phrase’s domain specificity. It assigned each candidate phrase a rank score by multiplying its within-document term frequency and domain-specificity score.

Different from the aforementioned approaches, we treat KE as a ranking problem and use the ranking SVM (rankSVM) approach [48] as it has been shown to be effective in KE in scientific literature, news, and weblogs [42].

Common learning features used by previous work include frequency-based features (eg, TF-IDF), term-related features (eg, the term itself, its position in a document, and its length), document structure-based features (eg, whether a term occurs in the title or abstract of a scientific paper), and syntactic features (eg, the part-of-speech [POS] tags). Features derived from external resources, such as Wikipedia and query logs, have also been used to represent term importance [39,40]. Unlike previous work, we explored rich semantic features specifically available to the medical domain.

Medelyan and Witten [45] developed a system that extends the widely used keyphrase extraction algorithm KEA [34] by using semantic information from domain-specific thesauri, which they called KEA++. KEA++ has been applied to the medical domain, where it used MeSH vocabulary to extract candidate phrases from medical articles and used MeSH concept relations to compute its domain-specific feature. In this study, we adapted KEA++ to the EHR data and used the adapted KEA++ as a strong baseline system.

## Methods

### A FOCUS Corpus of Electronic Health Records With Expert-Annotated Important Concepts

We created a FOCUS corpus, which is a collection of 90 representative EHR discharge summaries and progress notes from the University of Massachusetts Memorial Hospital outpatient clinics. To maximize the representativeness, we selected notes from patients with 6 different but common primary clinical diagnoses: cancer, chronic obstructive pulmonary disease, diabetes, heart failure, hypertension, and liver failure. We deidentified the notes and then asked physicians to identify, for each note, terms important to patients.

We adopted the expert annotation approach for this study for the following reasons. First, annotating important medical terms requires full comprehension of an EHR note. Such level of comprehension may be beyond the capacity of average patients [11-13,30]. Previous work shows that even lay people with higher education (ie, college or graduate degrees) have difficulty with comprehending EHR notes [11,30]. Second, physicians have specific medical training for communicating with patients and understanding their needs. Physicians' expertise would guide patients in understanding the most important aspects that are medically relevant to their health and well-being.

We developed an annotation guideline (see Multimedia Appendix 1) to instruct physicians to identify at least 5 of the most important medical terms per EHR note, which the patients need to know in order to comprehend the note for the most important aspects medically relevant to their health and treatment course. For each note, we obtained annotations from 2 physicians and used the agreement from both physicians as the gold standard for our experiments. Three physicians did the annotation and annotated 48, 68, and 64 notes, respectively.

### FOCUS

#### Overview

**Figure 1:**
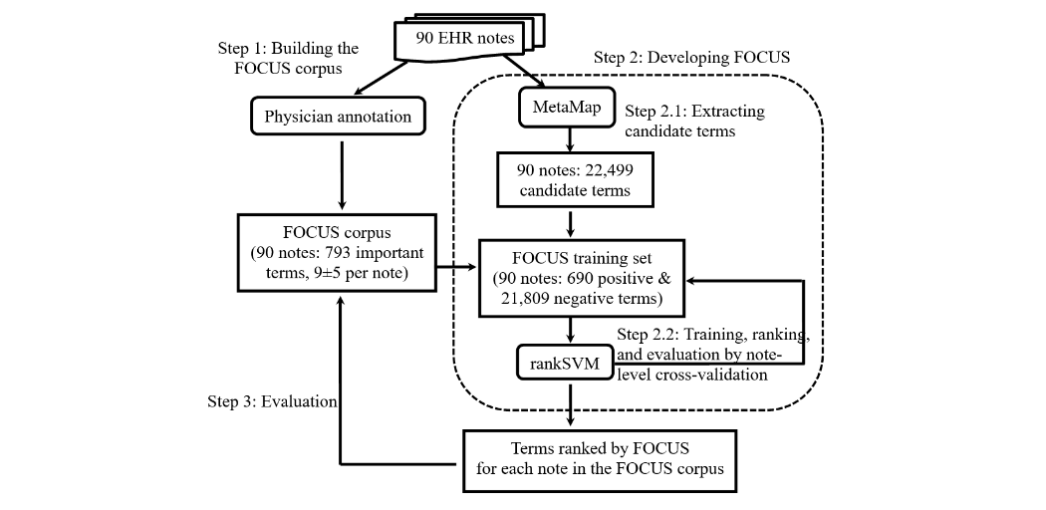
Overview of our approach: building the FOCUS corpus (Step 1), developing FOCUS (Step 2), and evaluation (Step 3). FOCUS: Finding impOrtant medical Concepts most Useful to patientS; EHR: electronic health record; rankSVM: ranking support vector machine.

Figure 1 shows the overview of FOCUS and its corpus and evaluation. In Step 2 of the approach, FOCUS first extracts candidate terms (Step 2.1) and then ranks them (Step 2.2). Since we focused on ranking in this study, we used MetaMap [29], a widely used medical concept detection tool, to automatically identify candidate terms from each EHR note. We then applied rankSVM to rank the terms.

#### Ranking Support Vector Machine

RankSVM [48] is a pairwise ranking method, which can learn to rank important terms in each EHR note as higher than nonimportant ones.

**Figure 2:**
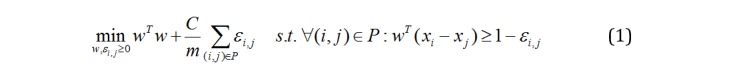
Objective function used in training ranking support vector machine.

Our training data for rankSVM contain the following: (1) a set *E* of EHR notes; (2) a list of candidate terms *T*_e_ associated with each EHR note *e*; and (3) for a term *t* ∊ *T*_e_, a *d*-dimension feature vector *x*_t_∊ *R*^d^and a binary target value (ie, label) *y*_t_ which denotes whether *t* is an important medical term in *e*. In our case, *y*_t_ is 1 if *t* is important in *e* and 0 if not. In the general framework of ranking, *y*_t_ corresponds to the ranking order of *t*, and the more important *t* is, the higher order and the larger value of *y*_t_ it has. Let *P* be the set of term pairs (*i, j*), where term *i* and term *j* occur in the same EHR note and term *i* is important (*y*_i_=1) and term *j* is not important (*y*_j_=0) (ie, *P*={ (*i, j*) | *y*_i_> *y*_j_}). The rankSVM model is built by minimizing the objective function [48], as defined by equation 1 in Figure 2, where *w* is the feature weight vector; *ε*_i,j_ is the slack variable that measures the model’s soft-margin error for term pair (*i, j*); *C* is a tuning parameter; and *m* is the total number of term pairs in *P*. The formulation in equation 1 in Figure 2 ﬁnds a large-margin linear function that minimizes the number of pairs of training examples swapped with respect to their desired ranking order.

We chose SVM^rank^[49], which implements rankSVM in an efficient way by using a cutting-plane algorithm and learns from large sparse data in linear time.

#### Baseline Features for Ranking

We implemented 9 features commonly used for KE [34,35,37,50,51].

##### Frequency-Based Features

The frequency-based features include term frequency, inverse document frequency, and TF-IDF. Term frequency is the number of occurrences of a candidate term in each individual EHR note. Inverse document frequency and TF-IDF are calculated in the standard way (see Multimedia Appendix 2). We used 6,237 clinical notes, which were selected by using the same 6 diagnoses used to select the 90 notes for the FOCUS corpus, to compute inverse document frequency.

##### Term Structure-Based Features

**Figure 3:**
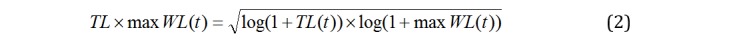
Equation for defining a combined feature of TL and maxWL. TL: term length (ie, length of a candidate term by word); maxWL: length of the longest word (by character) in a candidate term.

The term structure-based features include term length (TL) (ie, the total number of words contained in a term), the length of the longest word (by character) in a candidate term (maxWL), and a combined feature of TL and maxWL [51], as defined in equation 2 in Figure 3.

Since longer terms and words are less likely to be familiar to patients, these features may help distinguish between unfamiliar and common or familiar terms. Thus, these features may help rank as low EHR terms that are too common to be important (eg, *blood* and *pain*).

##### Position Feature

The position feature is the number of words preceding the first occurrence of a candidate term, normalized by the total number of words in the document. We used this feature because we found that the medical terms most specific to a patient often occur early in his/her EHR notes.

##### Lexical Feature

The lexical feature was found to be useful in domain-specific KE [35]. In our experiments, we used Porter’s stemmer to normalize terms. Since EHR data is noisy, we empirically include a stemmed term only if it occurs at least 3 times in the training data to eliminate misspelled words.

##### Part-of-Speech Feature

We used the POS tag of the head word of each candidate term, as generated by the clinical Text Analysis and Knowledge Extraction System (cTAKES) [52].

#### Additional Features for Ranking

##### Distributed Word Representation (Word Embedding)

Word embeddings are distributed vector representations of words learned from large unlabeled data. Words sharing similar semantics and context are expected to be close in their word vector space [53].

We include this feature because word embedding has emerged as a powerful technique for word representation. It has shown to improve several biomedical and clinical NLP tasks, such as biomedical named entity recognition [54,55], protein-protein interaction detection [56], biomedical event extraction [57,58], adverse drug event detection [59,60], ranking biomedical synonyms [61], and disambiguating clinical abbreviations [62,63].

We trained a neural language model to learn word embeddings. Specifically, we used Word2Vec software to create the skip-gram word embeddings [53,64]. We trained Word2Vec using a combined text corpus (over 3G words) of English Wikipedia, articles from PubMed Open Access, and 99,735 EHR notes from the Pittsburg corpus (Chapman W, University of Pittsburgh NLP Repository; using this data requires a license). We set the training parameters based on the study of Pyysalo et al [65]. We represented multi-word terms with the mean of individual word vectors. In this work, we used 200-dimension word vectors, with each dimension normalized to (0,1).

##### Unified Medical Language System Semantic Type

We mapped the candidate terms to Unified Medical Language System (UMLS) semantic types by using MetaMap, and included these semantic types as learning features.

##### Consumer Health Vocabulary Features

We derived 7 binary features from the consumer health vocabulary (CHV) [66]. The CHV is a collaborative resource and incorporates terms extracted from various consumer health sites, such as queries submitted to MedLinePlus and postings in health-focused online discussion forums [67-73]. The CHV contained 152,338 terms, most of which are consumer health terms [71-73]. Zeng et al [72] mapped these consumer health terms to the UMLS concepts by a semiautomatic approach. As a result of this work, the CHV encompasses lay terms as well as corresponding medical jargon.

In the FOCUS corpus, 89% of important terms are in the CHV, while a smaller percentage of nonimportant terms (76%) are in the CHV. This suggests that the presence of an EHR term in the CHV is indicative of the term’s importance from the perspective of patients (ie, health consumers). We therefore include a binary feature to denote whether a candidate term is in the CHV.

In addition, we derived 6 binary features from CHV familiarity scores. For extended usability, the CHV assigns familiarity scores to 57.89% (88,189/152,338) of its terms. CHV familiarity scores estimate the likelihood that a medical term can be understood by an average reader [74] and have values between 0 and 1, with 1 being most familiar and 0 being least familiar. CHV provides different types of familiarity scores [30]. Following Zeng-Treitler et al [30], we used the combined score and converted the continuous value into categorical features. Specifically, we divided the feature value range [0,1] into 5 equal-range bins, resulting in 5 binary features. The intuition behind these features is that medical terms with different levels of familiarity may be different in their importance to patients. For example, common terms (ie, terms that fall into the highest bin) such as *disease* and *physicians* are too general to be important. In addition, we included the sixth binary feature to indicate whether a candidate term has a CHV familiarity score.

##### Topic Features

**Figure 4:**
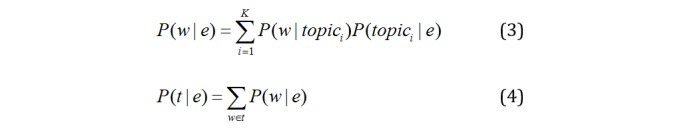
Equations for defining topic feature.

Topic features are real-valued features in (0,1) to indicate the topic coherence between a candidate term and the EHR note containing this term. We compute topic features *P(t|e)* by equations 3 and 4 in Figure 4, where *P(t|e)* is the probability of a candidate term *t* conditioned on an EHR note *e*; *P(w|e)* is the probability of a word *w* conditioned on *e*; *P* (*w* | *topic*_i_) and *P* (*topic*_i_ | *e*) are word-topic and topic-EHR note distributions estimated by the topic model; and *K* is the number of topics used in topic modeling.

We trained 3 latent Dirichlet allocation topic models with *K* set to 50, 100, and 200, respectively, after testing different *K*s on 6,237 clinical notes, which are the same as the notes used to compute IDF, using the MAchine Learning for LanguagE Toolkit (MALLET) [75] with default parameters to obtain 3 topic features.

### Training and Evaluation Settings

We created the training data from the FOCUS corpus as follows. We first applied MetaMap to the 90 notes in the FOCUS corpus. For each note, we took as positive examples those terms that were both identified by MetaMap and judged by physicians to be important to patients. We expanded the set of positive terms by using relaxed string match (details in the Evaluation Metrics subsection). The remaining terms identified by MetaMap were used as negative examples. This process resulted in a total of 690 positive and 21,809 negative terms from 90 notes.

Note that our 690 positive terms are less than the 793 terms annotated by physicians. This is because MetaMap missed some terms, many of which are multi-words with embedded UMLS concepts (eg, *autologous stem cell transplant* and *insulin-dependent diabetic*). Although we did not use these terms for training and for 10-fold cross-validation, we included them as positive terms for our final evaluation (as described in the Evaluation Metrics subsection).

We used the aforementioned training set for all the systems except 1 baseline system, adapted KEA++ (details in the Baseline Systems subsection), as it had its own procedure for extracting candidate terms and generating training data.

Previous work has shown that approximately 50-100 documents are sufficient to train supervised KE systems in the biomedical domain [45], suggesting that our 90 EHR notes, although a small size, may be sufficient. Our results empirically validated this hypothesis.

### Baseline Systems

#### Adapted KEA++

The keyphrase extraction algorithm KEA [34] has been frequently used as a strong baseline in previous work [42,43,47]. KEA++ [45] is an extension of KEA with the added capacity for domain adaptation.

KEA++ is based on naïve Bayes and uses the following 4 features: TF-IDF, term position, term length in words, and a knowledge-based feature node degree. The last feature computes the number of semantic links in a knowledge base that connect a candidate phrase to other phrases in the document. In addition, it supports preselection and filtering of candidate terms by using controlled vocabularies, which we adapted to the clinical vocabularies.

Specifically, we included all the UMLS terms identified by MetaMap from the 90 FOCUS notes. We also included the complete list of medical terms from 3 comprehensive clinical vocabularies: MeSH, Systematized Nomenclature of Medicine (SNOMED), and the ninth revision of the International Classification of Diseases (ICD-9). To compute the node degree feature, we mapped terms in this controlled vocabulary to the UMLS concepts and incorporated concept relations (eg, *Is-a* and *Part-of*) from MeSH, SNOMED, and ICD-9.

#### Random Forest

RF [76] is an ensemble learning method that combines multiple decision trees for classification or regression. RF extends the idea of *bagging* [77] with a random selection of features [78-80] to improve robustness and generalizability. The RF classification method achieved the state-of-the-art performance—outperforming KEA and kernel SVMs—in extracting keyphrases from scientific literature [43].

**Figure 5:**
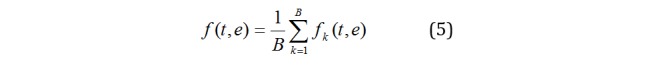
Prediction function of random forest.

We used the RF classification algorithm for our study. Assuming *t* is a candidate term from an EHR note *e*, the prediction of RF on (*t, e*), *ƒ*(*t,e*), is calculated by equation 5 in Figure 5, where *ƒ*_*k*_(*t,e*) is the prediction on (*t, e*) (ie, the predicted possibility of *t* being an important medical term in *e*) by the *k*th decision tree among *B* decision trees built for RF (see more details below). According to equation 5 in Figure 5, *ƒ*(*t,e*) represents the averaged predicted possibility of *t* being an important medical term in *e* and, therefore, can be used to rank candidate terms in *e*.

Each individual decision tree *ƒ_k_* is built as follows: assuming the training set contains *N* labeled examples (ie, *N* pairs of *t* and *e*, labeled as 1 if *t* is important in *e* and 0 if not) represented by *d* features, a single tree is built on *N* examples randomly sampled with replacement from this training set. When growing the tree, at each node the algorithm searches a randomly selected subset of the *d* features and selects 1 feature to create an if-then-else decision rule to branch the tree (ie, splitting the training examples at this node base on their feature values for the selected feature). Common criteria for selecting the feature that best splits a node include Gini impurity and information gain. When a node contains examples from the same class or its impurity is below a threshold, splitting stops and the node becomes a leaf node.

For a new example (*t, e*), RF assigns (*t, e*) to a leaf node of each individual decision tree by applying the decision rules learned from the training phase. The term *ƒ_k_*(*t,e*) in equation 5 in Figure 5 is calculated as the fraction of positive training examples in the leaf node of the *k*th decision tree where (*t, e*) is assigned.

RF uses the same features as FOCUS. We used scikit-learn [81] to develop RF. We set the parameter *B* by minimizing the out-of-bag error during training and used default values for other parameters.

### Evaluation Metrics

#### Precision, Recall, and *F*-score at Rank n

We report the averaged precision, recall, and *F*-score at ranks 5 and 10, abbreviated as P5, R5, and F5; and P10, R10, and F10, respectively. These metrics measure system performance for top ranks and are widely used to evaluate KE systems. We computed these metrics for the final evaluation (Step 3 in Figure 1) where we used all the gold-standard important terms as positive examples, including those that would never be included in the stage of candidate term extraction.

#### Area Under the Receiver Operating Characteristic Curve

Area under the receiver operating characteristic curve (AUC-ROC) is a metric widely used for evaluating ranking outputs. It computes the area under a receiver operating curve, which plots the true positive rate (y-coordinate) against the false positive rate (x-coordinate) at various threshold settings. To evaluate a system, we compute its AUC-ROC for each EHR note in the FOCUS corpus and report the averaged value. AUC-ROC measures the performance of the global ranking. Because both candidate term extraction and ranking affect the quality of global ranking, we report 2 AUC-ROC metrics: AUC-ROC_ranking_ and AUC-ROC_KE_. AUC-ROC_ranking_ is computed on the candidate terms extracted by a system. Thereby, if a gold-standard important term is missed in candidate term extraction, it will not affect the system’s AUC-ROC_ranking_. Since this metric is informative about the ranking performance of a system, we used it to evaluate the cross-validation results on ranking candidate terms (Step 2.2 in Figure 1). AUC-ROC_KE_ is computed by using all the gold-standard important terms as positive examples and measures the combined performance of candidate term extraction and ranking (Step 3 in Figure 1).

In the evaluation step, we use relaxed string match to determine true positives, as exact match is known to underestimate performance as perceived by human judges [50,82]. Specifically, we treat a term from the system output as a true positive if it either exactly matches or subsumes a gold-standard important term (eg, *non-Hodgkin lymphoma* subsumes *lymphoma*). We allow *subsume* but not *part-of* match in relaxed string match, as previous work found that the former aligned well with human judges but the latter did not [82]. For example, a part of an important term may be too general to be important (eg, *disease* in *Crohn's disease* and *iron* in *iron deficiency*).

### Statistical Analysis

The paired samples *t* test was used for significance testing for the performance difference of 2 systems.

## Results

### Statistics of FOCUS Corpus

For each note, we treat the terms agreed by 2 physicians as the gold-standard important terms. In total, the physicians have identified 793 important medical terms from the 90 FOCUS notes (mean 9 [SD 5] terms per note). The Cohen’s kappa coefficient for annotation agreement (microaverage) is .51. Table 1 summarizes the statistics of the FOCUS corpus.

The important terms identified by the physicians cover a wide range of topics, as represented by the UMLS semantic types. Table 2 shows term frequency and example terms for the 8 major topics.

**Table 1:** Statistics of the FOCUS^a^ corpus.

Characteristics of the FOCUS corpus	*N* or mean (SD)
Number of notes, *N*	90
Number of words per EHR^b^ note, mean (SD)	816 (133)
Number of candidate terms identified by MetaMap per EHR note, mean (SD)	250 (42)
Number of important medical terms identified by physicians per EHR note, mean (SD)	9 (5)

^a^FOCUS: Finding impOrtant medical Concepts most Useful to patientS.

^b^EHR: electronic health record.

**Table 2:** The 8 major topics in the FOCUS^a^ corpus.

UMLS^b^ semantic type	Number of important terms, *n*	Example terms
Disease or syndrome	295	autoimmune hemolytic anemia, gastroesophageal reflux, pancytopenia, Sjogren's syndrome, osteoporosis
Organic chemical	88	atenolol, vincristine, warfarin, Wellbutrin, Zocor
Finding	59	alopecia, hematuria, hypertension, NSTEMI (non-ST-elevation myocardial infarction), retinopathy
Neoplastic process	35	dermoid, large B cell lymphoma, pancreatic neoplasm, thyroid nodule
Therapeutic or preventive procedure	34	chemotherapy, dialysis, immunosuppression, kidney transplantation, pancreatectomy
Amino acid, peptide, or protein^c^	30	basal insulin, Rituxan, Neupogen, Synthroid, hemoglobin A1C, HPL (human placental lactogen)
Pathologic function	25	atrial fibrillation, autonomic dysfunction, BPH (benign prostatic hyperplasia), microscopic hematuria, systolic dysfunction
Diagnostic procedure	17	thyroid ultrasound, echocardiogram, endoscopy, biopsy, cardiac catheterization

^a^FOCUS: Finding impOrtant medical Concepts most Useful to patientS.

^b^UMLS: Unified Medical Language System.

^c^Electronic health record terms in this topic were split into 2 subtopics: medicine (denoted by their ingredients) and laboratory measure.

Most of the important terms annotated by physicians are specific to individual patients or notes. We used 2 criteria to select terms that may in general be important to patients: (1) the term occurs in more than 10% (9/90) of notes in the FOCUS corpus; and (2) the term was annotated as an important term for over 50% of the notes containing it. Only 4 terms were qualified and selected (the 2 bracketed numbers following the terms are the number of notes containing the term and the number of notes for which the term was annotated as important): *coronary artery disease* (20/14), *osteoarthritis* (19/10), *anemia* (13/7), and *prednisone* (10/6).

In addition, we made several observations from the FOCUS corpus. First, physicians typically excluded highly domain-specific terms that are very difficult for patients to understand. For example, the terms describing surgical procedures in detail or the anatomical parts of organs were excluded. Second, physicians often selected diseases and other information that are of immediate concern to patients, thus excluding other comorbidity diseases, for example. 

### Candidate Term Extraction

On average, adapted KEA++ extracts 342 candidate terms per note from the FOCUS corpus, which match 86% of the gold-standard physician annotated terms; FOCUS (the same for RF) extracts 250 candidates per note, which match 89% of the gold-standard terms.

### Evaluation on FOCUS Corpus

Table 3 shows the evaluation results on the FOCUS corpus, where FOCUS achieves the best results and RF is the second best.

The performance difference between FOCUS and adapted KEA++ is statistically significant for all the metrics (*P*<.001). The difference between FOCUS and RF is also statistically significant for all the metrics (see *P* values in Table 3).

**Table 3:** Performance of different natural language processing systems.

System	P5^a^	R5^b^	F5^c^	P10^d^	R10^e^	F10^f^	AUC-ROC_ranking_^g^	AUC-ROC_KE_^h^
Adapted KEA++^i^	0.333	0.211	0.239	0.281	0.362	0.292	0.890	0.780
RF^j^	0.409	0.267	0.299	0.339	0.416	0.346	0.891	0.821
FOCUS^k^	0.462	0.305	0.341	0.369	0.464	0.381	0.940	0.866
*P* (FOCUS vs RF)	.01	.01	.01	.045	.03	.02	<.001	<.001

^a^P5: precision at rank 5.

^b^R5: recall at rank 5.

^c^F5: *F*-score at rank 5.

^d^P10: precision at rank 10.

^e^R10: recall at rank 10.

^f^F10: *F*-score at rank 10.

^g^AUC-ROC_ranking_: area under the receiver operating characteristic curve computed on the candidate terms extracted by a system.

^h^AUC-ROC_KE_: area under the receiver operating characteristic curve (KE: keyphrase extraction) computed by using all the gold-standard important terms as positive examples.

^i^KEA++: extension of the keyphrase extraction algorithm KEA.

^j^RF: random forest.

^k^FOCUS: Finding impOrtant medical Concepts most Useful to patientS.

**Top-10 terms identified by different natural language processing systems for the full note containing the electronic health record excerpt in Textbox 1. True positives are italicized.**

Adapted KEA++: *Crohn disease*, cirrhosis, *metformin*, recent, iron deficiency, *thrombocytosis*, Crohn, *diabetes mellitus*, anemia, omeprazole

RF (random forest): cirrhosis, iron deficiency anemia, iron deficiency, *thrombocytosis*, fenofibrate, alcohol, cheilosis, *Crohn disease*, *myeloproliferative neoplasms, metformin*

FOCUS (Finding impOrtant medical Concepts most Useful to patientS): *thrombocytosis, diabetes mellitus,* cirrhosis, *diabetes, metformin,* omeprazole, iron deficiency anemia, fenofibrate, *Crohn disease, budesonide*

Textbox 2 shows the top-10 terms identified by each of the 3 systems for the full note containing the EHR excerpt in Textbox 1 (where true positives are italicized). The AUC-ROC_KE_ scores achieved by the 3 systems on the full note are 0.868 (FOCUS), 0.809 (adapted KEA++), and 0.857 (RF).

### Effects of Additional Features

We tested the effects of the additional features on FOCUS and RF. The results (see Table 4) show that the additional features improve the performances of both FOCUS and RF substantially (FOCUS vs FOCUS-base and RF vs RF-base). The difference is statistically significant for all the metrics except R10 between RF and RF-base.

We further tested the effect of each additional feature by adding it on FOCUS-base. The results (see Table A3-1 in Multimedia Appendix 3) show that each additional feature improves the baseline features to a certain degree.

We then tested FOCUS’s performance by using only additional features. The results (see Table A3-2 in Multimedia Appendix 3) show that word embedding is the best single feature, but still performs significantly worse than using all additional features for all the metrics (see row 5 in Table A3-2 in Multimedia Appendix 3 for *P* values). In addition, using only additional features performs significantly worse than using all features for all the metrics (*P*<.001).

**Table 4:** Performance of natural language processing systems with and without the additional features.

System	P5^a^	R5^b^	F5^c^	P10^d^	R10^e^	F10^f^	AUC-ROC_ranking_^g^	AUC-ROC_KE_^h^
FOCUS-base^i^	0.413	0.256	0.295	0.331	0.401	0.337	0.911	0.840
FOCUS^j^	0.462	0.305	0.341	0.369	0.464	0.381	0.940	0.866
*P* (FOCUS vs FOCUS-base)	.03	.02	.02	.003	<.001	.001	<.001	<.001
RF-base^k^	0.349	0.219	0.251	0.303	0.381	0.315	0.848	0.781
RF^l^	0.409	0.267	0.299	0.339	0.416	0.346	0.891	0.821
*P* (RF vs RF-base)	.003	.01	.01	.01	.10	.046	<.001	<.001

^a^P5: precision at rank 5.

^b^R5: recall at rank 5.

^c^F5: *F*-score at rank 5.

^d^P10: precision at rank 10.

^e^R10: recall at rank 10.

^f^F10: *F*-score at rank 10.

^g^AUC-ROC_ranking_: area under the receiver operating characteristic curve computed on the candidate terms extracted by a system.

^h^AUC-ROC_KE_: area under the receiver operating characteristic curve (KE: keyphrase extraction) computed by using all the gold-standard important terms as positive examples.

^i^FOCUS-base: Finding impOrtant medical Concepts most Useful to patientS; uses only the baseline features.

^j^FOCUS: Finding impOrtant medical Concepts most Useful to patientS; uses the baseline features plus the additional features.

^k^RF-base: random forest; uses only the baseline features.

^l^RF: random forest; uses the baseline features plus the additional features.

## Discussion

### Principal Findings

We have shown that physicians were able to identify important terms from EHR notes with moderate agreement (Cohen’s kappa .51). This level of annotation agreement is acceptable for keyphrase annotation tasks [40,42,83]. We used the physicians’ agreement to obtain high-quality data to develop and evaluate systems that automated this task.

Automated identification of EHR terms important to patients is challenging for several reasons. First, although frequency-based statistics such as term frequency and TF-IDF are widely used to estimate the importance of a term for a document, they are less effective for EHRs. For example, in our data, 56% of important medical terms occur only once in any individual EHR note. Second, we cannot infer the importance of a medical term solely based on its unfamiliarity level, as introduced in the Background and Significance subsection. Third, physicians’ annotations cannot be represented by simple patterns. One reason is that most patients in our data have comorbidity and the important terms identified by physicians are usually related to only some of their diseases. In addition, the important terms are spread over a wide range of topics—details in the Statistics of FOCUS Corpus subsection—and thus cannot be inferred by manual categorical rules. Fourth, EHR notes contain abundant medical terms, among which only a small portion (4% in our case) were annotated as positive or important. Such imbalanced data pose extra challenges for supervised learning.

Despite the above challenges, our FOCUS system achieves a decent 0.866 AUC-ROC, suggesting that the learning-to-rank model with rich features is effective.

### FOCUS Versus Adapted KEA++ and Random Forest

Our experiments show that FOCUS outperformed both adapted KEA++ and RF.

Using a more sophisticated MetaMap system, FOCUS is more effective than adapted KEA++ in candidate term extraction, as reported in the Candidate Term Extraction subsection. MetaMap is a state-of-the-art lexical tool that is well-configured—using morphological analysis and nonexact string match—to detect medical concepts and their corresponding medical terms from text, while adapted KEA++ uses a simpler approach (ie, dictionary look-up of stemmed *n*-grams from text).

We further compared FOCUS and adapted KEA++ on 28 FOCUS notes for which the 2 systems have the same recall on candidate extraction. FOCUS outperforms adapted KEA++ on this subset in all the evaluation measures, in particular, with significant improvements on AUC-ROC_ranking_(0.936 vs 0.903, *P*=.03) and AUC-ROC_KE_(0.875 vs 0.844, *P*=.03)_._ This indicates that the rich features and the rankSVM algorithm contribute to FOCUS’s performance gains.

Despite using the same MetaMap extractor and features, FOCUS still shows an advantage, outperforming RF in all the evaluation measures. The performance difference demonstrated that the ranking-based approach outperformed the state-of-the-art classification-based approach (RF) for this task. We attribute FOCUS’s advantage over RF to the rankSVM algorithm used by FOCUS. Specifically, rankSVM sets its parameters by minimizing the number of swapped pairs during its model training, which is equivalent to maximizing the rank quality as measured by Kendall’s tau coefficient. In contrast, the RF algorithm is based on decision trees. The rules guiding the construction of decision trees (eg, information gain) are not directly optimizing rank quality.

We further analyzed the top-10 terms identified by the 3 systems. FOCUS, RF, and adapted KEA++ respectively ranked 433, 417, and 379 unique terms in their top-10 lists—since we have 90 notes, the maximum number of unique terms is 900. This result indicates that all 3 systems output diversified top-ranked terms, which are not constrained by a small set of terms, with FOCUS’s output being the most diversified. We then identified terms frequently ranked as high (in the top 10) by each system using 2 criteria: (1) the term was identified as a candidate term for more than 10% (9/90) of the notes; and (2) the term was ranked in the top 10 over 60% of the time. The analysis results (see Table A4-1 in Multimedia Appendix 4) show that FOCUS and RF, RF and adapted KEA++, and FOCUS and adapted KEA++ share 6, 4, and 3 terms in their frequently ranked-as-high terms, respectively. Only 2 terms— *hypothyroidism* and *chemotherapy* —are frequently ranked as high by all 3 systems.

### Effects of Additional Features

Our additional features, when applied jointly, improved both FOCUS and RF (see Table 4). As FOCUS and RF adopt different learning schemes—ranking versus classification—these results suggest that the beneficial effect of our additional features is generalizable to different learning methods.

Among the additional features, word embedding improves the AUC-ROC scores most—these scores measure the quality of the global ranking (see row 2 in Table A3-1 in Multimedia Appendix 3). This feature has been successfully applied to other biomedical and clinical NLP tasks. To the best of our knowledge, our work is the first to apply word embedding to ranking important terms in EHRs and show its usefulness.

The UMLS semantic type is the best in boosting performance at top ranks (rank=5 and rank=10, row 3 in Table A3-1 in Multimedia Appendix 3), suggesting its importance. One reason why it is useful is that medical terms with certain semantic types such as *medical device* and *anatomical structure* were almost never annotated by physicians as being important to patients. This feature, therefore, can help rank those terms lower to improve quality of top ranks.

Although the 3 topic features only improve the baseline features slightly, further analysis shows that they, when combined with other features, improve the performance. In particular, the FOCUS system using complete features significantly outperformed the one not using the topic features on AUC-ROC (*P*=.03 for both AUC-ROC_ranking_ and AUC-ROC_KE_).

The FOCUS systems that respectively use only all additional features and only word embedding achieved adequate results, especially on AUC-ROC scores (see Table A3-2 in Multimedia Appendix 3). However, they still performed worse than the system using all features, especially at top ranks.

### Error Analysis and Future Work

We manually examined 17 notes, for which FOCUS has either zero recall at rank 5 or low AUC-ROC_KE_(<0.800). We identified 3 error patterns.

First, we used relaxed string match for evaluation but did not allow *part-of* match, for the reason discussed in the Evaluation Metrics subsection. However, in some cases, this approach underestimates the performance. For example, FOCUS counted it as a mistake if MetaMap recognized *stem cell transplant* but not *autologous stem cell transplant*, the gold-standard term.

Second, FOCUS depends on MetaMap, which makes mistakes. It failed to identify certain abbreviations as medical terms (eg, *A1c* [a lab test for blood glucose], *BMD* [a lab test for bone mineral density], *CPPD* [calcium pyrophosphate deposition disease], and *TSH* [a lab test for thyroid stimulating hormone]). In future work, we may collect a list of common clinical abbreviations by mining a large EHR corpus and use this list to enhance medical term identification.

Third, the error is due to data sparsity. Although word embedding helps overcome data sparsity, FOCUS failed to rank as high some infrequent medical terms, such as *femoral popliteal bypass* and *pseudogout*. In future work, we will explore advanced approaches to deal with out-of-vocabulary words.

### Limitations

Due to the common bottleneck of creating an expert-annotated resource, we only annotated 90 EHR notes for the reference standard and training data. Although this is not a large dataset, our system FOCUS shows an impressive performance of 0.940 AUC-ROC for 10-fold cross-validation on this data, suggesting that the data size may be sufficient.

### Conclusions

We have presented a new clinical NLP task—identifying medical terms important to patients from EHRs. We developed FOCUS, a learning-based NLP system that is based on SVM learning-to-rank algorithm and rich learning features. The evaluation done on 90 physician-annotated EHR notes showed that FOCUS significantly outperformed other state-of-the-art NLP systems and that the additional features we developed were beneficial in boosting its performance.

## Appendix

Multimedia Appendix 1 Guidelines for annotating medical terms important to patients in electronic health record notes.

Multimedia Appendix 2 Formulas for calculating frequency-based features.

Multimedia Appendix 3 Effects of additional features on FOCUS’s ranking performance. FOCUS: Finding impOrtant medical Concepts most Useful to patientS.

Multimedia Appendix 4 Medical terms frequently ranked as high by different natural language processing systems.

## Abbreviations

AUC-ROC: area under the receiver operating characteristic curve
BMD: bone mineral density
CBC: complete blood count
CHV: consumer health vocabulary
CPPD: calcium pyrophosphate deposition disease
cTAKES: clinical Text Analysis and Knowledge Extraction System
EHR: electronic health record
F5: *F*-score at rank 5
F10: *F*-score at rank 10
FOCUS: Finding impOrtant medical Concepts most Useful to patientS
ICD-9: ninth revision of the International Classification of Diseases
KE: keyphrase extraction
KEA: keyphrase extraction algorithm
KIP: keyphrase identification program
MALLET: MAchine Learning for LanguagE Toolkit
maxWL: length of the longest word (by character) in a candidate term
MeSH: Medical Subject Headings
NLP: natural language processing
P5: precision at rank 5
P10: precision at rank 10
POS: part of speech
R5: recall at rank 5
R10: recall at rank 10
rankSVM: ranking support vector machine
RF: random forest
SNOMED: Systematized Nomenclature of Medicine
SVM: support vector machine
TF-IDF: term frequency-inverse document frequency
TL: term length
TSH: thyroid stimulating hormone
UMLS: Unified Medical Language System
