# Supplementary material for: Finding Important Terms for Patients in Their Electronic Health Records: A Learning-to-Rank Approach Using Expert Annotations
Source: JMIR Med Inform. 2016 Nov 30;4(4):e40. doi: 10.2196/medinform.6373 (PMC5156821; doi:10.2196/medinform.6373)
Supplement: Multimedia Appendix 1 [file medinform_v4i4e40_app1.pdf]

Multimedia Appendix 1. Guidelines for annotating medical terms important to patients in electronic health record notes

1. Goal/task: identifying at least five most important medical terms per electronic health record (EHR) note which the patients need to know in order to better understand their EHR notes

In general, the goal can be achieved by selecting the minimum number of medical terms, which if the patients know, they will have a significant understanding of their clinical diseases and symptoms without being overwhelmed.

We provide operational rules in Section 2 to help achieve this goal.

2. Selection criteria
(1) Include terms that represent the main concept of each EHR note
Note: The most important medical terms that patients should know shall be straight-forward clinical knowledge, rather than complex clinical knowledge that may confuse patients or may need additional explanation

(2) Include terms that are related to the main concepts identified in (1) and can help patients' comprehension of the most important clinical concepts in their EHR notes

Note: These related terms also shall be straight-forward clinical knowledge, rather than complex clinical knowledge that may confuse patients or may need additional explanation

---

Notes:

The annotation guideline was developed by a group of physicians and non-physicians.

Textbox 1.1 shows an example annotation to illustrate "complex clinical knowledge that may confuse patients or may need additional explanation". The physicians did not annotate the terms "left axis deviation" and "nonspecific t-wave lowering", whose comprehension requires complex knowledge about EKG curve and heart function.

Textbox 1.1 An example annotation where terms judged by physicians to be important to patients are bracketed.

> Today's *EKG* shows <atrial fibrillation> with *controlled ventricular response*, *left axis deviation*, *nonspecific t-wave lowering* in many *leads*, and ongoing *fluctuation* since last year and the year before.
