# Supplementary material for: Finding Important Terms for Patients in Their Electronic Health Records: A Learning-to-Rank Approach Using Expert Annotations
Source: JMIR Med Inform. 2016 Nov 30;4(4):e40. doi: 10.2196/medinform.6373 (PMC5156821; doi:10.2196/medinform.6373)
Supplement: Multimedia Appendix 2 [file medinform_v4i4e40_app2.pdf]

Multimedia Appendix 2. Formulas for calculating frequency-based features

The frequency-based features include term frequency (TF), inverse document frequency (IDF) and TF-IDF. TF is the number of occurrences of a candidate term in each individual electronic health record (HER) note. IDF and TF-IDF are calculated in the standard way, as in (A2-1) and (A2-2):

$$IDF(t) = \log \frac{N}{\left| \left\{ e \middle| t \in e \right\} \right|} \qquad \text{(A2-1)}$$

$$TF - IDF(t) = TF(t) \times IDF(t) \qquad \text{(A2-2)}$$

where t is a candidate term; e is an EHR note; N is the total number of EHR notes in a data collection. We used 6K clinical notes (which were selected by using the same six diagnoses used to select the 90 notes for the FOCUS[a] corpus) to compute IDF.

[a]FOCUS: Finding impOrtant medical Concepts most Useful to patientS
