# Supplementary material for: Finding Important Terms for Patients in Their Electronic Health Records: A Learning-to-Rank Approach Using Expert Annotations
Source: JMIR Med Inform. 2016 Nov 30;4(4):e40. doi: 10.2196/medinform.6373 (PMC5156821; doi:10.2196/medinform.6373)
Supplement: Multimedia Appendix 3 [file medinform_v4i4e40_app3.pdf]

Multimedia Appendix 3. Effects of additional features on FOCUS's ranking performance

Table A3-1. Performances of different FOCUS[a] systems implemented by using baseline features or by adding each type of additional feature(s) on the baseline features. We report the *P* values (if *P*<.1) between each implementation and FOCUS-base[b].

System	P5[c]	R5[d]	F5[e]	P10[f]	R10[g]	F10[h]	AUC-$ROC_{ranking}$[i]	AUC-ROC_{KE}[j]
FOCUS-base	0.413	0.256	0.295	0.331	0.401	0.337	0.911	0.840
+ word embedding[k]	0.427	0.286	0.318	0.349	0.435 (*P*=.05)	*0.360 (P=.06)*	*0.933 (P<.001)*	*0.860 (P<.001)*
+ UMLS semantic type[l]	*0.456 (P=.03)*	*0.297 (P=.003)*	*0.335 (P=.005)*	*0.349 (P=.099)*	*0.438 (P=.01)*	0.359 (*P*=.04)	0.926 (*P*<.001)	0.853 (*P*<.001)
+ CHV[m]	0.433	0.271	0.310	0.338	0.409	0.343	0.919 (*P*=.003)	0.847 (*P*=.002)
+ topic[n]	0.418	0.261	0.300	0.349 (*P*=.08)	0.429 (*P*=.08)	0.358 (*P*=.06)	0.912	0.841

[a]FOCUS: Finding impOrtant medical Concepts most Useful to patientS.
[b]FOCUS-base: Finding impOrtant medical Concepts most Useful to patientS; uses only the baseline features.
[c]P5: precision at rank 5.
[d]R5: recall at rank 5.
[e]F5: F-score at rank 5.
[f]P10: precision at rank 10.
[g]R10: recall at rank 10.
[h]F10: F-score at rank 10.
[i]AUC-$ROC_{ranking}$: area under the receiver operating characteristic curve computed on the candidate terms extracted by a system.
[j]AUC-ROC_{KE}: area under the receiver operating characteristic curve (KE: keyphrase extraction) computed by using all the gold-standard important terms as positive examples.
[k]+ word embedding: FOCUS-base plus the word embedding feature.
[l]+ UMLS semantic type: FOCUS-base plus the unified medical language system semantic type feature.
[m]+ CHV: FOCUS-base plus the consumer health vocabulary features
[n]+ topic: FOCUS-base plus the topic features

Table A3-2. Performances of different FOCUS[a] systems implemented by using all features or by using only additional feature(s).

System	P5[b]	R5[c]	F5[d]	P10[e]	R10[f]	F10[g]	AUC-ROC$_{ranking}$[h]	AUC-ROC$_{KE}$[i]
FOCUS-full[j]	0.462	0.305	0.341	0.369	0.464	0.381	0.940	0.866
all additional features[k]	0.373	0.245	0.275	0.323	0.393	0.329	0.932	0.859
P (FOCUS-full vs. all additional features)	<.001	<.001	<.001	<.001	<.001	<.001	<.001	<.001
word embedding[l]	0.322	0.215	0.238	0.279	0.342	0.283	0.909	0.838
P (all additional features vs. word embedding)	.004	.02	.01	<.001	.001	<.001	<.001	<.001
UMLS semantic type[m]	0.229	0.144	0.165	0.220	0.280	0.228	0.873	0.803
CHV[n]	0.096	0.049	0.059	0.081	0.081	0.075	0.735	0.676
topic[o]	0.262	0.161	0.187	0.206	0.261	0.214	0.803	0.742

[a]FOCUS: Finding impOrtant medical Concepts most Useful to patientS.
[b]P5: precision at rank 5.
[c]R5: recall at rank 5.
[d]F5: F-score at rank 5.
[e]P10: precision at rank 10.
[f]R10: recall at rank 10.
[g]F10: F-score at rank 10.
[h]AUC-ROC$_{ranking}$: area under the receiver operating characteristic curve computed on the candidate terms extracted by a system.
[i]AUC-ROC$_{KE}$: area under the receiver operating characteristic curve (KE: keyphrase extraction) computed by using all the gold-standard important terms as positive examples.

[j]FOCUS-full: Finding impOrtant medical Concepts most Useful to patientS; uses all the features.
[k]all additional features: Finding impOrtant medical Concepts most Useful to patientS; uses all the additional features, including word embedding, the unified medical language system semantic type, the consumer health vocabulary features, and the topic features.
[l]word embedding: Finding impOrtant medical Concepts most Useful to patientS; uses only the word embedding feature.
[m]UMLS semantic type: Finding impOrtant medical Concepts most Useful to patientS; uses only the unified medical language system semantic type feature.
[n]CHV: Finding impOrtant medical Concepts most Useful to patientS; uses only the consumer health vocabulary features
[o]topic: Finding impOrtant medical Concepts most Useful to patientS; uses only the topic features
