# Supplementary material for: Finding Important Terms for Patients in Their Electronic Health Records: A Learning-to-Rank Approach Using Expert Annotations
Source: JMIR Med Inform. 2016 Nov 30;4(4):e40. doi: 10.2196/medinform.6373 (PMC5156821; doi:10.2196/medinform.6373)
Supplement: Multimedia Appendix 4 [file medinform_v4i4e40_app4.pdf]

Multimedia Appendix 4. Medical terms frequently ranked high by different natural language processing systems

Table A4-1. Terms frequently ranked high (in the top-10) by different natural language processing systems. We report three numbers in each cell for columns 2 to 4: the number of times the term is extracted as a candidate term by the system, the percentage of times the term is ranked in the top-10 by the system, and the percentage of times the system is correct when ranking the term in the top-10. Terms are ordered reversely by the first number.

Term	FOCUS[a]	RF[b]	Adapted KEA++[c]	Num. of times of being gold-standard[d]
hypertension	53|0.91|0.42	53|0.91|0.41		23
diabetes	31|0.81|0.48	31|0.81|0.40		13
hyperlipidemia	26|0.92|0.46	26|0.85|0.45		12
artery disease			21|0.81|0.00	0
coronary artery disease	19|1.00|0.68	19|0.84|0.69		14
osteoarthritis	18|0.89|0.50		16|0.69|0.82	10
lisinopril	17|0.65|0.09			6
tramadol		16|0.88|0.43		6
arthritis			15|0.67|0.00	1
lymphoma			14|0.93|0.08	1
GERD		13|0.87|0.38	15|0.67|0.60	7
anemia		12|0.75|0.67	21|0.76|0.25	7
hypothyroidism	12|0.83|0.50	12|0.75|0.56	11|0.82|0.44	5
pneumonia			12|0.67|0.00	1
prednisone			12|0.92|0.55	6
neuropathy	11|0.82|0.11			3
chemotherapy	10|0.80|0.38	10|1.00|0.30	12|1.00|0.25	3
cardiac catheterization			10|0.70|0.14	3
diabetes mellitus			10|0.80|0.38	3
vertigo			10|0.70|0.43	3
vitamin D deficiency		10|0.80|0.25		3

[a]FOCUS: Finding impOrtant medical Concepts most Useful to patientS.
[b]RF: random forest.
[c]KEA++: extension of the keyphrase extraction algorithm KEA.
[d]Num. of times of being gold-standard: the number of notes for which the term is a gold-standard important term.
